# PReS-FINAL-2270: Primary hypertrophic osteoarthropathy - a rare cause of swollen joints

**DOI:** 10.1186/1546-0096-11-S2-P260

**Published:** 2013-12-05

**Authors:** M Rodrigues, I Brito

**Affiliations:** 1 Pediatrics, São João Hospital, Porto, Portugal; 2 Pediatric Rheumatology Unit, São João Hospital, Porto, Portugal

## Introduction

Primary hypertrophic osteoarthropathy (PHO), also known as pachydermoperiostosis, is a rare genetic disease with excessive proliferation of skin and bone at the distal parts of extremities.

Features include clubbing of the digits, periostitis of the long bones, hydrarthrosis, facial skin thickening and coarse facial features. It mostly affects males and has a bimodal peak of onset: in the first year of life and around puberty.

Secondary hypertrophic osteoarthropathy is associated with an underlying pulmonary, cardiac, hepatic, or intestinal disease, and is rarely found in children.

## Objectives

A previously healthy 16 year-old adolescent was referred to the Pediatric Rheumatology Clinic with a one year history of painful and swollen knees, feet and arms. The pain was of a mechanical nature, with relief from NSAIDs, and no morning stiffness or nocturnal pain. He also complained about facial acne and palmoplantar hyperhidrosis. There were no constitutional symptoms, rashes, aphthae, Raynauds, ocular, gastrointestinal or respiratory complaints. Family history was irrelevant.

## Methods

On examination, there was clubbing of fingers and toes, hypertrophy of soft tissue (coarse facial features and thickening of the facial skin, with prominent folds on the forehead and cheek), cutaneous gland dysfunction (acne, hyperhydrosis, seborrhea) and joint swelling with effusion (knees, ankles).

Imaging showed soft tissue swelling and periosteal ossification with cortical thickening, metaphyseal diaphyseal enlargement of long bones, with preservation of articular surfaces, and no acroosteolysis. Bone scanning (Tc-99m) revealed symmetrically increased uptake in the tubular bones along the cortical margins of the diaphysis and metaphysis (parallel-track sign).

ESR and CRP were mildly elevated, with normal endocrine workup, full blood count and film, renal and hepatic function, LDH, electrolytes and bone biochemistry, except for low 25-OH vitamin D. Autoantibodies and immunology were normal. Chest X-Ray, Mantoux test, Abdominopelvic US and cardiac evaluation had no changes.

## Results

NSAID treatment was successful in managing the pain. After 1-year of follow-up, there are no new complaints, no signs of a secondary cause. A referral to Genetics was made, and he is currently awaiting testing.

## Conclusion

PHO is a rare cause of joint swelling and can be confounded with other causes of polyarthritis.

Careful physical examination will detect digital clubbing and raise the diagnostic suspicion.

Exclusion of secondary causes is paramount, as is close clinical follow-up as some patients eventually develop diseases many years later.

Although its course is self-limiting, and progression stops at the end of adolescence, there is no curative treatment for the skeletal abnormalities.

## Disclosure of interest

None declared.

